# Quality of Longer Term Mental Health Facilities in Europe: Validation of the Quality Indicator for Rehabilitative Care against Service Users’ Views

**DOI:** 10.1371/journal.pone.0038070

**Published:** 2012-06-04

**Authors:** Helen Killaspy, Sarah White, Christine Wright, Tatiana L. Taylor, Penny Turton, Thomas Kallert, Mirjam Schuster, Jorge A. Cervilla, Paulette Brangier, Jiri Raboch, Lucie Kalisova, Georgi Onchev, Spiridon Alexiev, Roberto Mezzina, Pina Ridente, Durk Wiersma, Ellen Visser, Andrzej Kiejna, Patryk Piotrowski, Dimitris Ploumpidis, Fragiskos Gonidakis, José Miguel Caldas-de-Almeida, Graça Cardoso, Michael King

**Affiliations:** 1 Mental Health Sciences Unit, University College London, London, United Kingdom; 2 Division of Population Health Sciences and Education, St George’s University London, London, United Kingdom; 3 Department of Psychiatry, Psychosomatic Medicine and Psychotherapy, Park Hospital Leipzig, Leipzig, Germany; 4 University Hospital Department of Psychiatry and Psychotherapy, Dresden University of Technology, Dresden, Germany; 5 San Cecilio University Hospital Mental Health Unit, University of Granada, Granada, Spain; 6 Biomedical Research Centre Mental Health Network, University of Granada, Granada, Spain; 7 Department of Psychiatry, Charles University, Prague, Czech Republic; 8 Department of Psychiatry, Medical University Sofia, Sofia, Bulgaria; 9 Department of Mental Health, Trieste Healthcare Agency, Trieste, Italy; 10 University Medical Center Groningen, University of Groningen, Groningen, The Netherlands; 11 Department of Psychiatry, Wroclaw Medical University, Wroclaw, Poland; 12 University Mental Health Research Institute, Athens, Greece; 13 Faculty of Medical Science, New University of Lisbon, Lisbon, Portugal; University of Western Brittany, France

## Abstract

**Background:**

The Quality Indicator for Rehabilitative Care (QuIRC) is a staff rated, international toolkit that assesses care in longer term hospital and community based mental health facilities. The QuIRC was developed from review of the international literature, an international Delphi exercise with over 400 service users, practitioners, carers and advocates from ten European countries at different stages of deinstitutionalisation, and review of the care standards in these countries. It can be completed in under an hour by the facility manager and has robust content validity, acceptability and inter-rater reliability. In this study, we investigated the internal validity of the QuIRC. Our aim was to identify the QuIRC domains of care that independently predicted better service user experiences of care.

**Method:**

At least 20 units providing longer term care for adults with severe mental illness were recruited in each of ten European countries. Service users completed standardised measures of their experiences of care, quality of life, autonomy and the unit’s therapeutic milieu. Unit managers completed the QuIRC. Multilevel modelling allowed analysis of associations between service user ratings as dependent variables with unit QuIRC domain ratings as independent variables.

**Results:**

1750/2495 (70%) users and the managers of 213 units from across ten European countries participated. QuIRC ratings were positively associated with service users’ autonomy and experiences of care. Associations between QuIRC ratings and service users’ ratings of their quality of life and the unit’s therapeutic milieu were explained by service user characteristics (age, diagnosis and functioning). A hypothetical 10% increase in QuIRC rating resulted in a clinically meaningful improvement in autonomy.

**Conclusions:**

Ratings of the quality of longer term mental health facilities made by service managers were positively associated with service users’ autonomy and experiences of care. Interventions that improve quality of care in these settings may promote service users’ autonomy.

## Introduction

Despite the move towards community based mental health care in Europe over recent decades, many patients still reside in some form of institution [1]. Although the exact number of longer term mental health facilities is unknown, concerns have been raised about the continuing reliance on large asylums in less economically developed countries [2] and the expansion of the “virtual asylum” of smaller health and social care facilities provided by the independent sector in countries with better developed community mental health services, catering to service users with more complex needs [3]. These facilities absorb a large proportion of the mental health budget of most countries and concerns about the quality of care provided have been raised [2], [4]. A review of studies that investigated the costs of care associated with deinstitutionalisation in England, Germany and Italy concluded that well managed community based care was more cost-effective than long-stay hospital care as it was able to provide better quality care that resulted in better clinical outcomes [5].

The people who reside in longer term facilities mostly have diagnoses of psychotic illnesses [6] with complications such as treatment resistance [7], cognitive impairment and negative symptoms [8], poor social functioning [9], substance misuse and challenging behaviours [10]. They are at risk of abuse of their human rights since their capacity to make informed choices and participate actively in their care may be impaired. The European Commission’s Green Paper [11] on improving the mental health of the population specifically highlighted the promotion of social inclusion for this group, protection of their fundamental rights and dignity. However, until recently, there were no standardised measures available to assess the quality of care in longer term mental health facilities. The Quality Indicator for Rehabilitative Care (QuIRC) was developed to address this gap through a study funded by the European Commission involving ten European countries at different stages of deinstitutionalisation (Bulgaria, Czech Republic, Germany, Greece, Italy, Netherlands, Poland, Portugal, Spain and the UK) [12].

The QuIRC assesses seven domains of care in longer term hospital and community mental health units (Living Environment; Therapeutic Environment; Treatments and Interventions; Self-Management and Autonomy; Social Interface; Human Rights; and Recovery Based Practice). The domains of care included in the toolkit were identified through triangulation of the results of: i) a review of care standards in each country; ii) a systematic literature review of the components of care (and their effectiveness) in longer term mental health units [13]; iii) an international Delphi exercise with four stakeholder groups in each of the ten countries, involving 447 participants (service users, carers, professionals, advocates) [14]. The toolkit collects data on various aspects of the unit including: staffing; staff training and supervision; built environment; treatments and interventions offered; availability of activities for service users; care planning processes; involvement of service users in their own care and the running of the unit; promotion of service users’ autonomy, independence and physical health; policies and processes relating to managing challenging behavior; facilitation of service users’ access to and involvement in community activities; involvement of families and carers; policies and processes related to complaints and confidentiality; facilitation of service users’ access to advocacy and legal representation. The toolkit was piloted and refined with input from an international expert panel. Its inter-rater reliability was then tested in 202 units and found to be excellent [15]. The final version comprises 145 questions that can be completed by the unit manager in less than one hour. Of these, 86 items contribute to scores on the seven QuIRC domains. A web based version of the QuIRC [16] provides a printable report on the unit’s performance on the seven domains, presented as percentages for ease of interpretation by unit managers. Further details of the content of the QuIRC, its item structure and psychometric properties are published elsewhere [15].

Since the toolkit assess the quality of a facility from information provided by the unit manager, we also aimed to validate the QuIRC ratings by investigating their association with service users’ ratings of the quality of care [12], [15]. This paper reports on the results of this validation. Inter-rater reliability of unit manager and service user QuIRC ratings was not feasible since the QuIRC was designed for completion by the unit manager and contains many items that service users would not have been able to answer. Instead service users’ assessments of the quality of care were made using standardised measures of their experiences of care, quality of life, autonomy and assessment of the facility’s therapeutic milieu.

## Method

### Ethics Statement

The study was approved by the relevant ethics committees in each of the ten participating countries involved in developing the QuIRC (Bulgaria - Ethics Committee, Alexandrovska University Hospital; Czech Republic - General University Hospital, Prague, Ethics Committee; Germany – Ethik Kommission der Medizinischen Fakultät Carl Gustav Carus an der Technischen Universität Dresden; Greece - University Mental Health Research Institutes Medical; Italy - Comitato Etico Indipendente; the Netherlands - Medical Ethical Committee of the University Medical Centre; Poland - Commission of Bioethics, Wroclaw Medical University; Portugal - Ethical Committee of the New University of Lisbon Medical School; Spain - Comisión Etica de la Universidad de Granada; UK - City and East London Multi Region Ethics Committee).

### Recruitment

At least 20 units that provided longer term care (at least six months) for adults with severe mental health problems were recruited in each of the ten countries. Units had to provide for at least six patients/residents, have communal facilities and staff on site, 24 hours per day. Units that only provided for other, specific groups (such as those with learning disability, organic brain injuries, substance misuse or dementia) were excluded. Hospital and community based units were recruited to give a range in size and geographical spread within countries. Sampling was not random; units were identified from registration lists in each country and/or were known to the lead investigator in each country. After gaining informed consent, the manager of each unit was interviewed using the QuIRC by the researcher in the relevant country.

A list of each unit’s current service users was generated by the unit manager. Service users were randomly selected for potential participation from each unit with a recruitment target range of between five and 13 per unit; five was agreed by the study partners as the minimum required for a representative sample and 13 was agreed an appropriate maximum since additional participants would not add further data about that unit relevant to the study aims. In units with 13 or fewer beds, all service users were approached for potential participation. In larger units, random sampling was carried out by the research team in each country; each service user on the unit manager’s list was allocated a number and a random number generator programme distributed by the lead centre (University College London) was used to identify those who the researcher should approach for potential participation. Written informed consent was then gained by the researcher before proceeding with a face to face research interview. Where fewer than five service users were recruited the unit was excluded and a further unit recruited. Service user participants were paid 10 Euros for their time in all countries except Bulgaria where such payments were not usual practice. Data were collected between February and September 2009.

### Service User Measures

For each of the four standardised measures used to assess service users’ assessment of the quality of care, higher scores represented better experiences. Quality of life was assessed using the Manchester Short Assessment of Quality of Life (MANSA) [17] which has been translated for use in many European countries. The service user rates 12 aspects of their life on a scale from 1 (couldn’t be worse) to 7 (couldn’t be better) and a total mean score between 1 and 7 is generated. The Resident Choice Scale (RCS) [18] was used to assess service users’ experiences of autonomy in the unit; the freedom to choose from a range of options without any coercion to bias that choice. Although there are no measures developed specifically for the assessment of autonomy of people with long term mental health problems, the issues relevant to those in longer term mental health facilities relate to mental capacity and the degree to which the facility promotes freedom of choice and independence across all aspects of everyday living. These aspects are captured in the RCS which required only minor adaptation for our purposes (the deletion of four items). The service user rates the degree to which they have choice over various aspects of daily activities (e.g. meal times) and the running of the unit on a four point scale (“I have no choice at all about this”, “I have very little choice about this”, “I can express a choice about this but I do not have the final say”, “I have complete choice about this”). A total score with a range 22 to 88 is generated. The degree to which service users felt involved in their treatment and care was assessed using the Your Treatment and Care (YTC) [19] questionnaire which has been used in the UK in service user led assessments of mental health services. The service user is asked to rate 25 items related to their care (e.g. “I know who my Doctor is”) as “yes, “no” or “don’t know”. The number of “yes” answers is summed to give a total score with a possible maximum of 25. The Good Milieu Index (GMI) [20] is a five item scale that was used to assess the unit’s therapeutic culture from the service user’s perspective. Service users rate their general satisfaction with the unit, with staff and other residents, and the degree to which they feel the unit facilitates their confidence and abilities on a scale of one to five (from “not at all” to “very much”) and a total score ranging from 5 to 25 is generated. An assessment of service user function was also made by the researcher using the Global Assessment of Function (GAF) [21] in order to take this into account as a potential mediator. All measures were translated and back translated in each centre and checked for accuracy of content at the lead centre. Researchers were trained in the use of all measures by HK. Inter-rater reliability of GAF scores was assessed at a training session for all researchers from each centre (a total of 20) using clinical vignettes and found to be 0.88 (95% CI: 0.76, 0.96).

### Data Management and Analysis

A common SPSS database was developed in the lead centre and distributed to all centres. A test entry of pilot data in each centre clarified any coding queries. Double data entry was completed for 10% of the toolkit data using a separate database and the study statistician (SW) carried out data validation on the two databases for each centre. The maximum error rate was set at 5%. Any centre that had an error rate above this was required to complete double data entry for all their data.

A multilevel model was used for the analysis of associations between QuIRC ratings and service user ratings with the aim of identifying the domains of care that independently predicted better service user experiences of care. Multilevel modelling allowed analysis of associations between service user ratings (level 1 data) as dependent variables with unit QuIRC domain ratings (level 2 data) as independent variables. To be able to test for 10 predictors of a medium effect size (R^2^ = 0.35) with 90% power at a 1.25% significance level (as four dependent variables were explored), a minimum of 203 level 2 units were required [22]. The predictor variables were the seven QuIRC domain ratings plus an overall QuIRC score - the sum of all 86 individual items scored in the seven domains. Unit and service user variables which needed to be controlled for as potential mediators were agreed by the research partners (community or hospital based unit, service users’ age, diagnosis of psychosis or not, and level of functioning as assessed by GAF) and included in the models.

The four service user (level 1, dependent) variables (MANSA, RCS, YTC and GMI) were all normally distributed. Associations with the eight unit (level 2, independent) variables, also normally distributed, were investigated (the seven QuIRC domains and the total QuIRC score). The QuIRC domain ratings and the total score were correlated with each other (21 out of the 28 pair wise correlations were above 0.5, nine correlations were above 0.8) so could not be entered simultaneously into regression models and were therefore entered separately.

Three sets of models were then fitted: in *Model A* only the indicated domain score was entered as an independent variable, a fixed effect. A random intercept term was included to adjust for the multiple service users per unit; in *Model B* the level 2 unit type variable (hospital or community) was added to *Model A*, along with the interaction between domain score and unit type; in *Model C* three level 1 service user characteristic variables were entered - age, GAF score and diagnosis (psychosis or not), in addition to the domain score and unit type. In addition to the random intercept term, random slopes were also included for age and GAF score. The interaction term added in *Model B* was removed in *Model C* as it was non-significant in all models. To illustrate the relationships found in the models the percentage of mental health unit-to-unit variation in the respective dependent variable explained by each of three models, *A, B* and *C* is presented. The *B-A* values represent the amount of extra variation explained by the inclusion of the unit type variable, *C-B* values show the amount of extra variation explained by the inclusion of the level 1 service user characteristic variables. *Model C* was only fitted when the domain score was significantly related to the dependent variable. In all models a country random effect was included.

## Results

### Response

A total of 213 units participated in the study of which 109 (51%) were in the inner city, 67 (32%) in the suburbs and 37 (17%) in a rural location. The majority (131, 62%) were in the community, 45 (21%) were hospital wards and 37 (17%) were units within the hospital grounds. Their size ranged from five to 120 beds (mean 26, median 18) and 31 (15%) units were for men only and 19 (9%) for women only. Overall, 2495 service users were randomly sampled for potential participation in the study of whom 722 (29%) were unable to give informed consent for the research interview, 23 (1%) declined to participate and 1750 (70%) were interviewed (two of whom had data missing for age). Service users were recruited from each country as follows: Bulgaria 180; Czech Republic 171; Germany 189; Greece 150; Italy 179; Netherlands 175; Poland 176; Portugal 170; Spain 210; UK 150.

### Service User Characteristics

Of the 1750 service users, over one third (651, 37%) were residing in a hospital ward and the rest were coded as “community” for the purposes of our analysis. Almost two thirds (1087, 62%) were male, the mean age was 46 years (range 18 to 87), most were unemployed (547, 31%) or retired (906, 52%), with only 50 (3%) in paid employment. Two thirds (1173/1750, 67%) had a diagnosis of psychosis and the mean length of stay in the current unit was 277 weeks (median 129, SD 838). The mean (SD) GAF score was 49 (15) and ranged from 20 to 80. In most countries, data on participants who were approached but did not agree to participate were not gathered in accordance with the guidance from the relevant ethics committee. However, data were available on 193 of the 745 non-participants; they did not differ from participants in mean age, gender or diagnosis (psychosis or not).

No centres had data entry error rates over 5% and therefore double data entry was not required.

### Association between QuIRC Domain Scores and Service User Ratings

Table 1 shows the “percentage of variation explained” statistic for each of the eight independent variables and four dependent variables. Examining the *Model A* results row for each independent variable, the following can be seen: over 10% of the unit-to-unit variation in service users’ mean quality of life (MANSA) scores was explained by the Living Environment and Self-Management and Autonomy domains of the QuIRC; 55% of the unit-to-unit variation in service users’ mean autonomy (RCS) scores was explained by the Self-Management and Autonomy domain, and overall QuIRC score, Living Environment, Recovery Based Practice and Human Rights domains each explained 30–35% of the explainable variation; over 35% of the unit-to-unit variation in service users’ mean experiences of care (YTC) scores was explained by the Self-Management and Autonomy domain and overall QuIRC score, and the Living Environment, Recovery Based Practice, Human Rights and Therapeutic Environment domains each explained 20–28%. The Self-Management and Autonomy domain explained 23% of the unit-to-unit variation in service users’ mean scores of therapeutic milieu (GMI) and 16% was explained by the Living Environment domain. The Social Interface and Treatments and Interventions domains explained very little variation in any of the dependent variables.

**Table 1:** Percentage of unit-to-unit variance in service user outcomes explained by QuIRC domain scores using three models.

	Model	Quality of Life(MANSA)	Autonomy (RCS)	Experience of care(YTC)	Therapeutic Milieu(GMI)
**QuIRC total**	A	0.1	31.2	35.6	12.1
	B	1.7	40.6	38.2	17.4
	B-A	1.6	9.4	2.6	5.3
	C		35.7	29.1	36.0
	C–B		−4.9	−9.1	18.6
**Therapeutic Environment**	A	−1.0	17.2	19.8	4.8
	B	3.1	34.0	28.5	13.2
	B–A	4.1	16.7	8.7	8.4
	C		26.9	19.4	34.3
	C–B		−7.1	−9.1	21.0
**Treatments and Interventions**	A	−0.6	11.9	15.7	1.9
	B	3.5	28.8	24.4	10.1
	B–A	4.1	16.9	8.6	8.3
	C		22.0	15.2	
	C–B		−6.8	−9.1	
**Human Rights**	A	2.8	31.6	24.8	9.9
	B	3.6	40.3	28.5	16.8
	B–A	0.8	8.7	3.7	6.8
	C	10.5	39.3	21.6	40.8
	C–B	6.8	−1.0	−6.9	24.1
**Recovery Based Practice**	A	3.5	30.1	28.3	15.7
	B	2.8	39.6	31.5	20.0
	B–A	−0.7	9.5	3.2	4.3
	C	9.8	33.9	21.7	41.5
	C–B	7.0	−5.7	−9.8	21.5
**Social Interface**	A	0.1	6.4	6.8	3.0
	B	3.9	26.0	18.1	14.7
	B–A	3.7	19.6	11.2	11.7
	C		17.9	7.1	
	C–B		−8.1	−11.0	
**Self-Management and Autonomy**	A	10.9	55.1	36.7	23.0
	B	9.1	56.4	35.9	25.0
	B–A	−1.8	1.2	−0.8	2.1
	C	16.8	51.9	27.5	43.8
	C–B	7.7	−4.4	−8.3	18.8
**Living Environment**	A	11.3	35.7	27.2	16.2
	B	9.3	36.3	26.2	17.8
	B–A	−2.0	0.5	−1.0	1.6
	C	15.5	35.9	19.6	32.6
	C–B	6.2	−0.4	−6.6	14.7

QuIRC  =  Quality Indicator for Rehabilitative Care.

Model A: QuIRC domain score entered as the only independent variable.

Model B: unit type (hospital or community) added to Model A.

Model C: service user characteristics added to Model B (age, GAF and psychosis or not).

Differences in % variance for each model also shown: B–A, C–B.

### Summary of Results for Each Dependent Variable

#### Quality of life (MANSA)

In *Model A,* overall QuIRC score, Therapeutic Environment, Treatments and Interventions and Social Interface domain scores were not found to be associated with service users’ quality of life. Whilst the other four domains were significantly associated with quality of life, Living Environment and Self-Management and Autonomy each explained only approximately 11% of unit-to-unit variation, and Recovery Based Practice and Human Rights each explained only 3–4%. Adding in type of unit in *Model B* made little or no difference to these results. In *Model C*, quality of life was found to be highly influenced by service user characteristics being included in the model, with approximately 30% more variation being explained by their inclusion. For each of the four domains explored in *Model C* (Human Rights, Recovery Based Practice, Self-Management and Autonomy, Living Environment), age, GAF and diagnosis were associated with quality of life as main effects but no interactions were significant. Age and GAF were positively associated with quality of life; those with a psychotic disorder having a slightly higher quality of life.

In summary, while there was evidence that staff ratings of their units’ Living Environment and promotion of Self-Management and Autonomy explained some of the variation in service users’ quality of life between units, service users’ characteristics had a greater influence on this.

#### Autonomy (RCS)

All QuIRC domain scores and overall QuIRC score were significantly associated with service users’ autonomy in *Model A*. The Self-Management and Autonomy domain score explained most of the unit-to-unit variation (55%) and the Social Interface domain explained the least (6%). Adding in the type of unit in *Model B* resulted in 9–20% more variation being explained for all domains except Self-Management and Autonomy and Living Environment. This suggests that these domains contain items which are highly related to unit type. Adding in service user characteristics in *Model C* did not result in further explanation of unit-to-unit variation. Diagnosis was not associated with autonomy. Age and GAF were significant as main effects but had few significant interactions on domain scores. Age was negatively associated with autonomy (younger people scoring higher) and GAF score was positively associated (better functioning was associated with higher autonomy scores). There was a significant interaction between GAF and Living Environment when modelled. The slope of the association for Living Environment scores and autonomy was greater for those with lower GAF scores. In other words, the association between the quality of the unit’s Living Environment and its service users’ autonomy was greater for those with poorer functioning.

In summary, all QuIRC domain scores were highly related to service users’ autonomy, particularly the Self-Management and Autonomy domain. The type of unit was also important, users in hospital units having significantly lower levels of autonomy. User characteristics did not explain further variation between the units but age and GAF were significantly associated with autonomy.

#### Experiences of care (YTC)

In *Model A*, all QuIRC domains were significantly associated with service users’ experiences of care, with the Self-Management and Autonomy domain and overall QuIRC score each explaining over one third of the unit-to-unit variation in YTC score. The Social Interface domain explained the least variation (7%). Adding in type of unit in *Model B* increased the percentage of variation explained in all but the Self-Management and Autonomy and Living Environment domains, although this effect was minimal for the overall QuIRC score, Human Rights and Recovery Based Practice domains. For other domains (Therapeutic Environment, Treatments and Interventions, Social Interface) there was an association between type of unit and experiences of care, with service users in hospital units having lower ratings on these three domains. For all domains, age and GAF were associated with experiences of care as main effects but few interactions were significant. Diagnosis was not associated with experiences of care. Age was negatively associated with experiences of care, younger people scoring higher, and GAF was positively associated with experiences of care, with better functioning being associated with higher scores. When Social Interface was modelled the slope of the association with experiences of care was higher for older service users (borderline significant).

In summary, all QuIRC domain scores were highly related to service users’ experience of care, particularly the Self-Management and Autonomy domain and the overall QuIRC score. There was some evidence that service users in hospital units had poorer experiences of care than those in community units. Service user characteristics did not explain further variation between the units but age and GAF were significantly associated with experiences of care.

#### Therapeutic milieu (GMI)

In *Model A*, all QuIRC domain scores, apart from Social Interface and Treatments and Interventions were significantly associated with the therapeutic milieu of the unit. The Recovery Based Practice, Living Environment and Self-Management and Autonomy domains explained the most unit-to unit variation (16–23%). Adding in unit type in *Model B* increased the amount of variation explained between units for all domains apart from Self-Management and Autonomy and Living Environment. Service users in hospital units had lower GMI ratings (by just over half of one point). Adding in service user characteristics in *Model C* increased the amount of between unit variation explained in the models by between 15 and 24%, suggesting a strong relationship between service user characteristics and GMI, independent of domain scores and type of unit. The GAF scores and age were both positively associated with GMI, though diagnosis was not. However, some of the interactions between diagnosis and domain scores were significant when modelled and the slope (strength) of the associations between the Recovery Based Practice, Therapeutic Environment and overall QuIRC scores and GMI score was greater for those without a psychotic disorder.

In summary, the Self-Management and Autonomy and Living Environment domain scores explained most of the variation in service users’ ratings of units’ therapeutic milieu, and the Social Interface and Treatments and Interventions domains were not associated with therapeutic milieu. Service users’ ratings of therapeutic milieu were highly influenced by service user characteristics, with older and better functioning service users rating the GMI higher.

### Clinical Relevance

In order to illustrate the clinical relevance of changes in domain scores (change in quality of care), we calculated the impact of a 10% increase in each domain score on service users’ autonomy and experiences of care. These two service user measures were chosen as they were not influenced by service user characteristics. A change of three points on the autonomy scale (RCS) was equivalent to either having complete choice on an issue (item) that the service user originally had no choice at all, or moving one point along the scale towards increased choice on three different items. On the measure of experiences of care (YTC) a change of one point was equivalent to answering ‘Yes’ to one further item of the 25 in this tool.

Table 2 shows the degree to which autonomy and experiences of care scores would be improved by a 10% improvement in each domain score. Results are shown for all units and for community units over hospital units.

**Table 2:** Estimated change in service users’ autonomy and experiences of care given an increase of 10% in each QuIRC domain.

QuIRC domain		Autonomy Score(RCS)		Experience ofCare Score(YTC)	
		Mean Change	Significance	Mean Change	Significance
**Therapeutic Environment**	**All units**	4.2	<0.001	0.9	<0.001
	**Community** **vs hospital**	7.5	<0.001	1.1	0.003
**Treatments and Interventions**	**All units**	3.8	<0.001	0.9	<0.001
	**Community** **vs hospital**	7.5	<0.001	1.1	0.004
**Human Rights**	**All units**	4.1	<0.001	0.8	<0.001
	**Community** **vs hospital**	6.2	<0.001	0.8	0.026
**Recovery Based Practice**	**All units**	3.8	<0.001	0.7	<0.001
	**Community** **vs hospital**	5.4	<0.001	0.6	0.090
**Social Interface**	**All units**	1.5	0.001	0.3	<0.001
	**Community** **vs hospital**	7.8	<0.001	1.1	0.004
**Self-Management and Autonomy**	**All units**	4.3	<0.001	0.8	<0.001
	**Community** **vs hospital**	2.1	0.065	0.2	0.588
**Living Environment**	**All units**	3.3	<0.001	0.6	<0.001
	**Community** **vs hospital**	2.1	0.147	0.1	0.891

QuIRC  =  Quality Indicator for Rehabilitative Care.

RCS  =  Resident Choice Scale.

YTC  =  Your Treatment and Care.

A 10% improvement in any QuIRC domain score, except Social Interface, was associated with a statistically and clinically significant increase in service users’ autonomy scores of at least three points. This effect was greater for service users in community based units than those in hospital based units for all domains except Self-Management and Autonomy, and Living Environment.

A 10% improvement in any QuIRC domain score was associated with a statistically significant increase in service users’ experience of care scores of 0.3 to 1.1 points. The effect of these improvements was greater for service users in community based units compared to hospital units for all domains except Recovery Based Practice, Self-Management and Autonomy, and Living Environment.

## Discussion

We found direct links between the quality of an institution (QuIRC domains) and its service users’ experiences of care and autonomy. All QuIRC domains except Treatments and Interventions and Social Interface were found to be significantly positively associated with service users’ assessments of the units’ therapeutic milieu, though service user characteristics accounted for most of this association. The QuIRC domains Living Environment and Self-Management and Autonomy were significantly positively associated with service users’ quality of life but again, service user characteristics accounted for much of the association. The associations between QuIRC domain scores and service user autonomy and experiences of care were independent of service user characteristics.

Autonomy is the freedom to choose from a range of options without any coercion to bias that choice. However, it may be affected by mental incapacity secondary to mental illness [23]. Our findings are particularly relevant therefore, since the associations we found between quality of care as assessed by QuIRC and service user autonomy were not mediated by service user function. These findings give confidence that the unit quality ratings derived from the unit manager concurred with service users’ experience of the care provided and the degree to which the unit promoted their autonomy. In developing a new assessment tool, the usual approach to validation is to assess its convergence against an existing measure that assesses a similar construct, or against expert opinion. However this was not possible since there was no measure assessing the quality of longer term mental health institutions available, and expert opinion is usually used for clinical assessment tools. Given that the QuIRC is completed by the manager of the facility, we felt it was appropriate to assess its association with the experiences of care of those using the service. In other words, our results provide further validation of the toolkit domain ratings.

Ideally, staff and service users should be interviewed when assessing the quality of a facility, but in situations where service user interviews are not feasible (for example, where service users are too unwell to participate or lack capacity to give informed consent to do so), our findings suggest that the QuIRC ratings derived from the unit manager may provide a proxy indication of the overall service user experience of care and autonomy in that unit.

We demonstrated that a hypothetical, small increase in any QuIRC domain quality rating (of 10%) resulted in improvements in service user autonomy and experiences of care. This effect appeared to be more clinically meaningful for service user autonomy than experiences of care. This suggests that initiatives to improve unit quality could potentially benefit service users in achieving greater autonomy, one of the main aims of contemporary mental health services [24]. The effect on service user autonomy appeared generally greater for those in community based, rather than hospital based, units. However, increase in quality in the Living Environment and Self-Management and Autonomy domains was not associated with a significant improvement in the autonomy of service users of community based units. This may reflect a “ceiling effect” since community based facilities have been shown to be less “institutionalised” than hospital settings [25], [26].

Whilst our study included over 200 units from ten countries at different stages of deinstitutionalisation across Europe, we did not randomly sample units for potential participation and therefore those that took part may not be representative of other longer term mental health units of the countries that were involved. Whilst this did not pose any systematic bias relevant to the purpose of our study (to assess the internal validity of the QuIRC), we are mindful that it is relevant to its external validity. For example, units that were willing to participate may be of higher quality than other units. However, one centre (Portugal) included all its longer term units in this study and the QuIRC has since been used to assess the quality of all mental health rehabilitation units in England without problem.

We randomly identified service users for participation in order to minimise selection bias, but almost one third were unable to give informed consent to participate. Our findings could therefore be subject to response bias since those who were least well were unable to be interviewed. However, our analyses took account of service users’ global functioning in order to mitigate against this potential limitation and service user characteristics were not found to influence the associations we found between unit quality and service users’ autonomy and experiences of care. The high non-participation rate due to lack of capacity also highlights the need for proxy assessments in this service user group.

As in all cross-sectional observational studies, our results remain open to residual confounding. For example, other, unmeasured user characteristics may have affected the associations we observed or obscured others we missed. Nevertheless, the positive associations we found between the quality of the unit and the service user experience not only support the validity of QuIRC, but also provide helpful indications for how care might be improved for the large number of people whose mental health problems necessitate their residence in longer term facilities across Europe.

In conclusion, ratings of the quality of longer term mental health facilities made by service managers using the QuIRC were positively associated with service users’ ratings of their autonomy and experiences of care. In situations where service user interviews are not feasible, the QuIRC may provide a proxy indication of the overall service user experience. Interventions that improve quality of care in these settings may promote service users’ autonomy.
